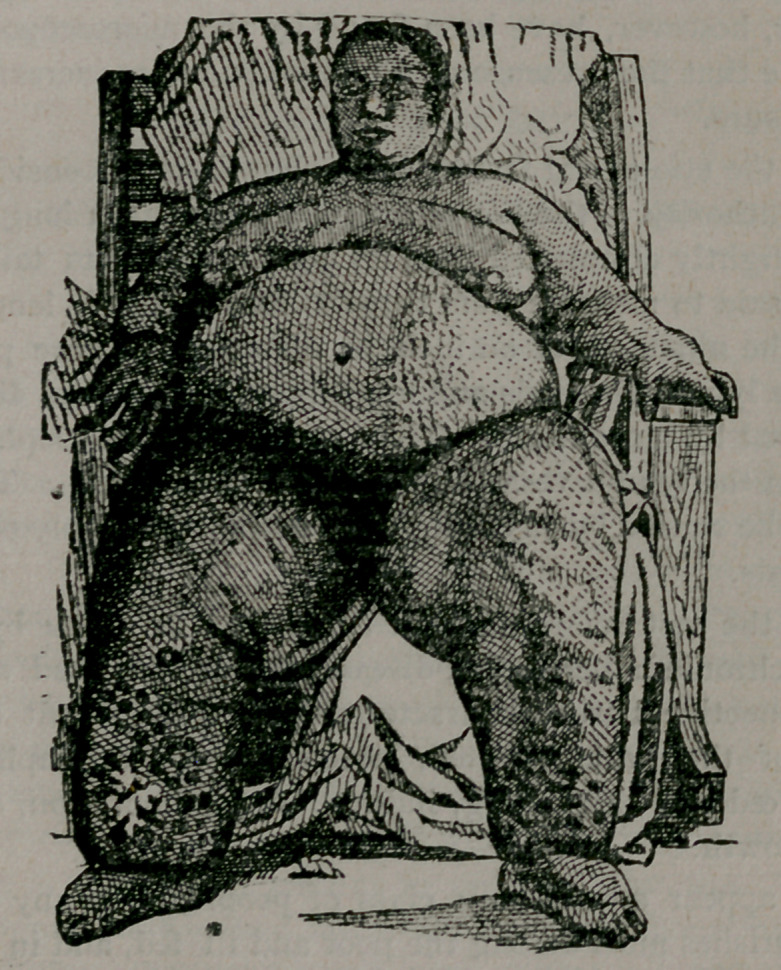# A Remarkable Case of Elephantiasis

**Published:** 1889-08

**Authors:** T. J. Bennett

**Affiliations:** Austin, Texas


### A REMARKABLE CASE OF ELEPHANTIASIS.

BY T. J. BENNETT, M. D., AUSTIN, TEXAS.

Read before the Austin District Medical Society, June 20, 1889.

MATILDE S., age 50, colored female, resident of Austin for thirty years. Family history good. Patient is from a family several members of which weigh considerably over 200 pounds each, and patient herself has weighed over 400 pounds for the last five or ten years.

Fifteen years ago the disease with which the patient is afflicted, began to develop near the ankles. It appeared first, as do a great many cases of elephantiasis, in the form of erysipelas—a mere inflammation of the skin and subcutaneous tissue. This attack was treated and apparently cured, but in a few months a second attack set up in the same locality, from which the patient did not entirely recover, the integument remaining somewhat thickened and indurated. Repeated attacks of this inflammation continued to recur every few months for a number of years. Each invasion left the parts more and more indurated, thickened and roughened, until the whole of the lower extremity became enormously enlarged. There are several large ulcers on the lower part of the leg, both on the posterior and anterior surfaces. The hypertrophic skin and enlarged lymph vessels are not confined, as is usual in this disease, to the lower limbs, but nearly the whole body is more or less affected. On the abdomen and anterior aspect of the shoulders and chest are numerous corrugations and ridges as large as the little finger. The cut does not bring them out in full. A patient, naturally as corpulent as this one is, to be thus affected, presents a figure rarely observed.

The dimensions of the patient are as follows: Height, 5 feet 3 inches; weight, 440 pounds; ankle, 26½ inches in circumference; knee, 31½ inches; thigh, 46 inches; abdomen, 6 feet; largest part 6 feet 6½ inches.

I may state that these measurements were taken after the patient had been considerably reduced by the free use of tincture of digitalis and the bitartrate of patassa, which remedy the patient has remained upon four or five weeks, and has been greatly benefited so far as the distension is concerned.

At oue time death appeared imminent from dyspucea, due to the great distension of the skin, lymph vessels and subcutaneous connective tissues. The patient has been compelled to sleep in a half erect position for months.

Albumen has been present in the urine in considerable quantity since I first saw the patient, about four months ago. No tube cysts, however, have been found by the microscope, and it is probable that the presence of albumen is due to increased vascular pressure.

One of the etiological factors in elephantiasis, as considered by eminent authority, is the *filaria sanguinis hominis*, a long slender parasite slightly attenuated from its head towards its tail. The female is said to measure from three to four inches in length, and is about the nineteeth of an inch in diameter. These parasites live in the lymph channels and are supposed to be first deposited in the blood by mosquitoes. They are a cause of elephantiasis only when the lymph vessels are obstructed by them. The disease may be caused by anything that partially or wholy obstructs these canals.

Oue of the best definitions of elephantiasis is given by Dühring: "A chronic hypertrophic disease of the skin and subcutaneous connective tissue, characterized by enlargement and deformity of the parts affected, accompanied by lymphangitis, swelling, œdema, thickening, induration, pigmentation, and papillary growth."

It may appear among any class of people, or in any climate, but it flourishes most among the poor and ill fed, and in a warm climate. It is claimed by a recent writer that the natural habitation of elephantiasis is the Samoan islands.

Its treatment is very unsatisfactory. Amputation of affected limbs, ligation of arteries, etc., have been practiced with only ameliorating results at best. Constitutional treatment has been of no avail further than mitigating of symptoms, and in some instances, prolonging of life.

The chief interest in the case above reported lies in the unusual extent of surface involved which includes nearly three-fourths of the entire body. The case is reported mainly on account of elephantiasis being a rare disease in this part of the country. This is the only case recorded in the State so far, as I am aware.

NOTE.—Since writing the above the patient has died, apparently from the effects of over-distention of the lymph vessels and connective tissues. No autopsy.

## Figures and Tables

**Figure f1:**